# Elusive variants in autosomal recessive disease: how can we improve timely diagnosis?

**DOI:** 10.1038/s41431-023-01293-0

**Published:** 2023-02-03

**Authors:** Ari E. Horton, Sebastian Lunke, Simon Sadedin, Andrew P. Fennell, Zornitza Stark

**Affiliations:** 1 Monash Genetics, Monash Health, Melbourne, VIC Australia; 2 Monash Heart and Monash Children’s Hospital, Monash Health, Melbourne, VIC Australia; 3 Monash Cardiovascular Research Centre, Victorian Heart Institute, Melbourne, VIC Australia; 4 Department of Paediatrics, Monash University, Melbourne, VIC Australia; 5 Victorian Clinical Genetics Services, Murdoch Children’s Research Institute, Melbourne, VIC Australia; 6 University of Melbourne, Melbourne, VIC Australia; 7 Australian Genomics, Melbourne, VIC Australia

**Keywords:** Medical genomics, Genetic testing

Although an estimated 80% of rare diseases have a genetic origin [1], and rare disease accounts for 15.9% of hospital admissions [2], a molecular diagnosis remains elusive in >50% of individuals with a suspected genetic disorder [3]. Typically, the first-line genomic investigation for rare disease remains exome sequencing, due to its’ relative cost-efficiency and established practices for data analysis and interpretation of coding variants. However, a proportion of elusive diagnoses have molecular aetiologies that are not tractable by exome sequencing [4], raising questions about how and when other technologies such as short- and long-read genome sequencing, transcriptome and proteomic analyses should be integrated into diagnostic pathways.

We reflect on this in the context of being involved in the care of two siblings with Schimke immuno-osseous dysplasia (SIOD), for whom molecular diagnosis still took 3 years despite early access to ultra-rapid exome sequencing (Fig. 1). The proband, a 3-year-old female, presented with poorly responsive steroid-resistant nephrotic syndrome and focal segmental glomerulosclerosis, rapidly progressing to end-stage renal disease. She was enrolled in the Australian Genomics Acute Care Genomics study and underwent ultra-rapid trio exome sequencing [5], which identified a single likely pathogenic variant in *SMARCAL1*, which is associated with autosomal recessive SIOD with a time to report of 3.8 days (Fig. 2). No second copy number variant was identified on high-resolution chromosomal microarray. She progressed to develop immunodeficiency and bone marrow failure, increasing the clinical suspicion of SIOD. Meanwhile, her younger sibling/brother presented at age 3 with short stature, microcephaly (<1st centile) and mild proteinuria, without immunodeficiency.

**Fig. 1:**
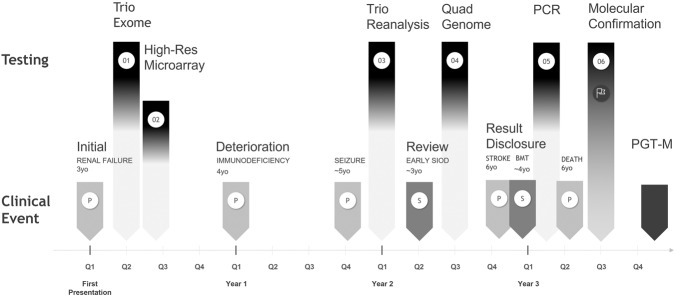
Timeline – Clinical Events and Diagnostic Testing. P: proband, S: sibling, high-res high resolution, BMT: bone marrow transplant, PGT-M: pre-implantation genetic testing, SIOD: Schimke immuno-osseous dysplasia.

**Fig. 2:**
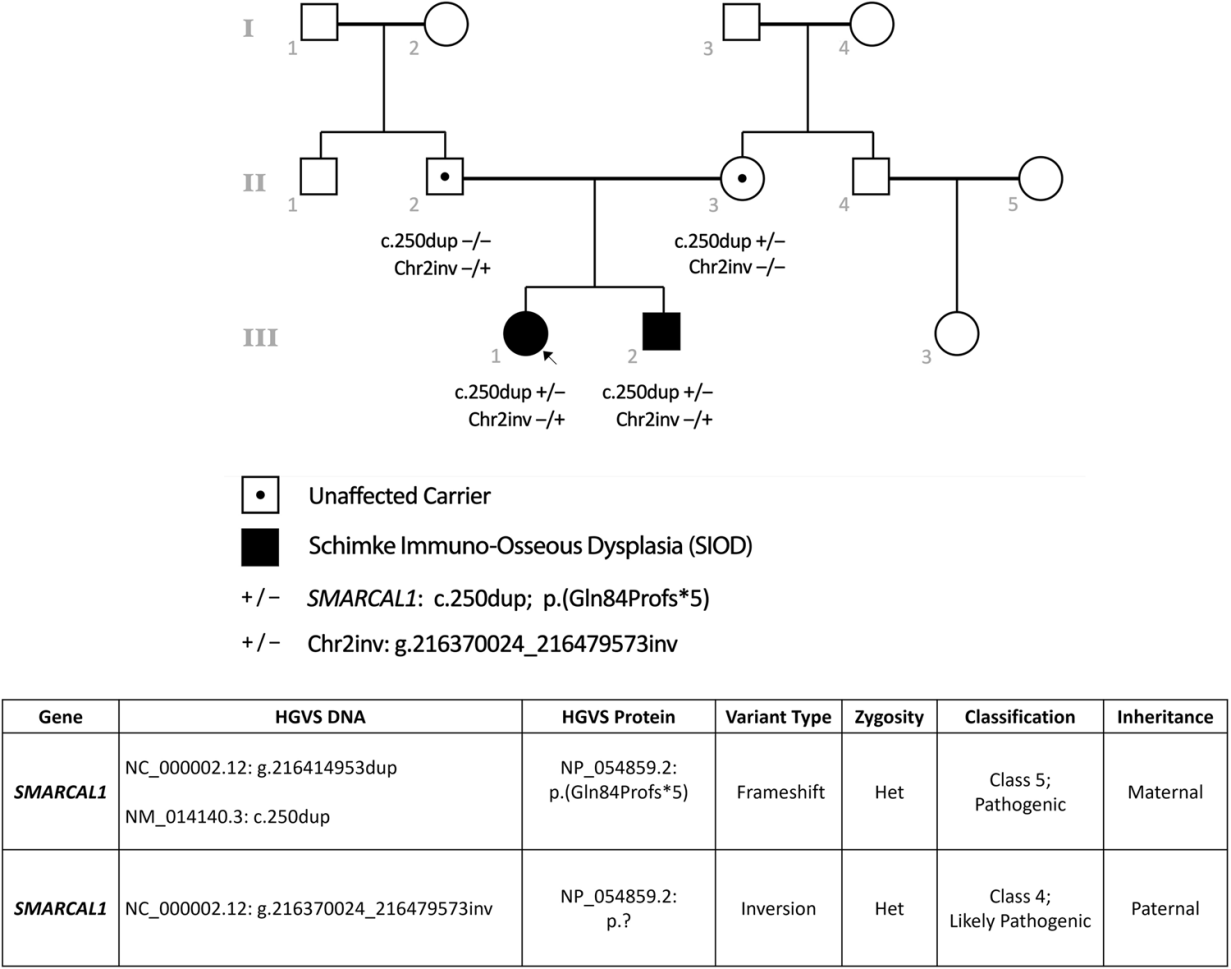
Family pedigree and biallelic variants in *SMARCAL1*. Het – heterozygous.

Genome sequencing was accessed through the research study and was performed in the parents and both affected siblings. Manual inspection of the WGS data identified a paternally inherited chromosome 2 rearrangement including a suspected inversion in the region of *SMARCAL1*. This was orthogonally confirmed by PCR analysis at the putative breakpoints, finally establishing a firm molecular diagnosis of SIOD in both siblings after 3 years (Fig. 3). The diagnosis came too late for the older sibling, who sadly died at age 6 years due to complications of SIOD. The younger sibling underwent pre-symptomatic bone marrow transplant with a good outcome. Importantly, unlocking the molecular diagnosis allowed the parents to undergo pre-implantation genetic testing for both *SMARCAL1* variants to achieve an unaffected pregnancy.

**Fig. 3:**
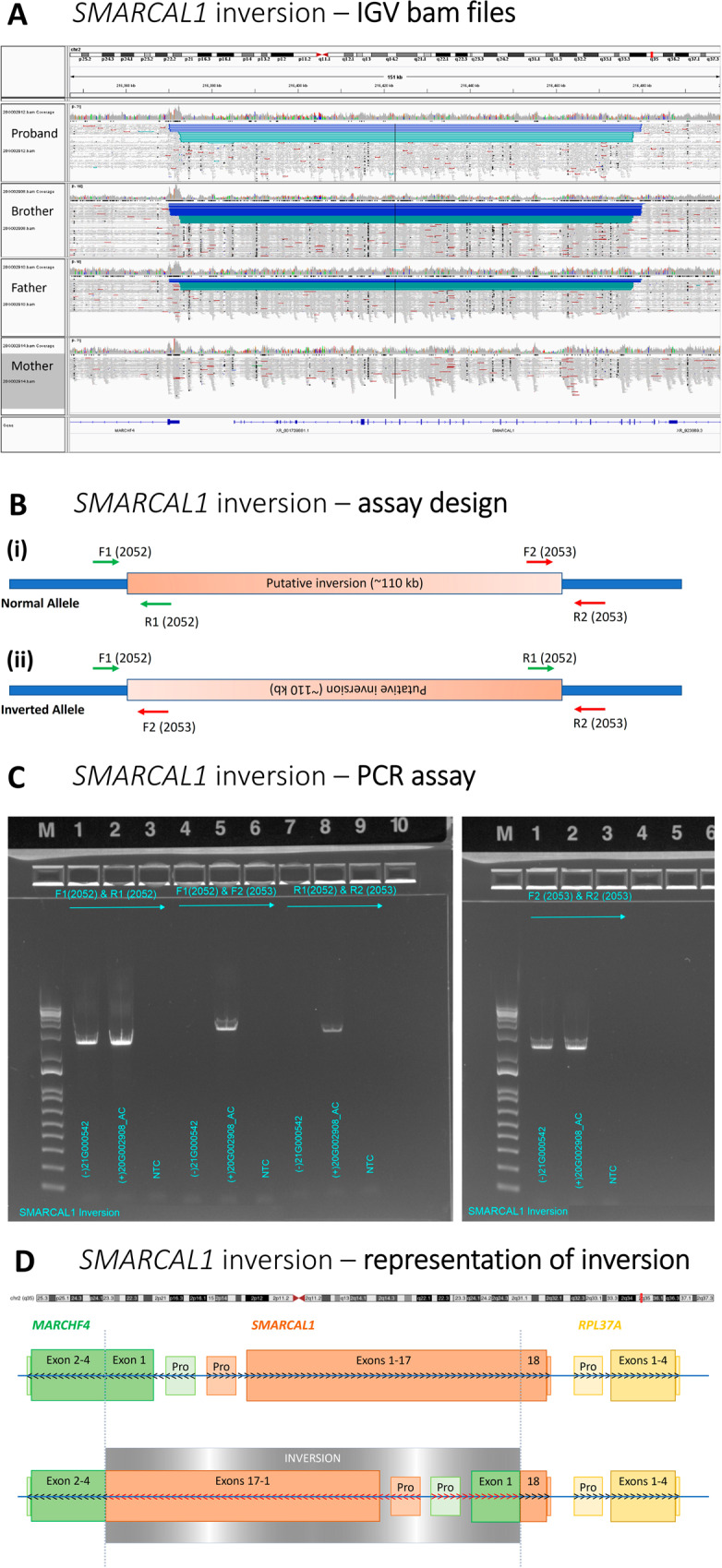
Detection and confirmation of chromosome 2 inversion involving *SMARCAL1*. **A** Integrative Genomics Viewer (IGV) WCR-free WGS read data visualisation showing the four family members and the site of the inversion; **B** Assay design for orthogonal confirmation demonstrating the inversion; **C** PCR assay demonstrating the presence or absence of inversion breakpoints in the family members; **D** Representative diagram demonstrating the inversion involving Exons 1–17 (of 18) of SMARCAL1 and the 5′UTR and beginning of exon 1 (p.1–79) of MARCHF4; Pro – Promoter region of corresponding gene; arrows indicate the standard 5′–3′ read strand of each gene, black depicts standard with red depicting the reversed direction of the gene after the structural variant, actual reading frame of the fusion gene may differ.

Deciding between first-line exome sequencing or genome sequencing remains a hot topic for discussion. Cases like this highlight the potential utility of first-line genome sequencing, with the ability to detect multiple variant types in a single test, resulting in significant reductions in time to diagnosis and affording potential healthcare cost savings together with the opportunity to intervene early and improve clinical outcomes [6]. When exome sequencing has already been performed and no diagnosis has been achieved, there is lack of guidance regarding if, and when genome sequencing should be considered as a second-tier test, and attendant lack of funded pathways. We highlight the group of patients with suspected autosomal recessive disorders, where one pathogenic variant has already been identified, as particularly likely to benefit from second-tier genome sequencing. *ABCA4*-associated Stargardt disease, a relatively common inherited retinal disease, provides a clear model for identification of missing heritability in autosomal recessive disorders. In a cohort of 67 individuals with clinically diagnosed Stargardt disease, with either one (*n* = 64) or no (*n* = 3) variants identified, Bauwens et al used the systematic application of testing technologies and bioinformatics analyses to identify a diagnosis in 83%. The missing heritability was accounted for by a range of variant types such as novel (deep-) intronic splice, cis-regulatory, structural, and recurrent hypomorphic variants. An integrated approach combining genomics with downstream tailored functional studies allows not only molecular diagnosis but also the opportunity for personalised therapies in the future.

These studies illustrate some of the other practical considerations that need to be addressed in the quest to improve diagnostic outcomes in routine practice. Copy number variants, structural variants, and short tandem repeats require specialised bioinformatic analyses for detection, and these may or may not be part of clinically accredited analysis pipelines [7]. Even when detected, these variant types are more likely to require custom-designed orthogonal validation. Clinical interpretation remains challenging due to the lack of guidance for determining whether these variants are pathogenic. As an example, population databases such as gnomAD and DGV currently have limited data for structural rearrangements. Variant classification guidelines including ACMG/AMP and classification of constitutional copy number variants [8, 9] may not be applicable in these variant types. This may result in classification as a variant of uncertain significance, requiring ancillary studies (including RNA studies) to establish downstream effects and pathogenicity [10]. Establishing pathways to effectively detect, validate and interpret a range of variant types in the routine diagnostic setting will be required to achieve diagnoses in a timely and cost-effective manner.

The added clinical utility of confirming a molecular diagnosis can be significant. In this case, the diagnostic trajectory took 3 years and 4 months from first presentation to a confirmed molecular diagnosis (Fig. 1). Employing a genome sequencing first approach may have altered the clinical trajectory of the proband, facilitating early bone marrow and renal transplantation, which may have resulted in a different clinical outcome.

Future advances in diagnostic genomics have the potential to further transform the ability to achieve a molecular diagnosis. The continuing evolution of long-read sequencing technologies such as PacBio and Oxford Nanopore, together with future implementation of the Telomere-to-Telomere (T2T) genome, have the potential to unlock vast swathes of the genome that have been hitherto uninterpretable, including complex rearrangements, repeat segments, and pericentromeric regions. In the future, bridging technical and translational gaps will undoubtedly have a major impact on the timeliness of rare disease diagnostics. Together with the increasing availability of targeted therapeutics, this brings us closer to realising the potential of the genomic revolution.

## Data Availability

The data that support the findings of this study are available on request from the corresponding author. The data are not publicly available due to privacy or ethical restrictions.
